# Wounding, insect chewing and phloem sap feeding differentially alter the leaf proteome of potato, *Solanum tuberosum* L.

**DOI:** 10.1186/1477-5956-10-73

**Published:** 2012-12-26

**Authors:** Marc-Olivier Duceppe, Conrad Cloutier, Dominique Michaud

**Affiliations:** 1 Département de phytologie/Centre de recherche en horticulture, Pavillon des services (INAF), Université Laval, Québec, QC, G1V 0A6, Canada; 2 Département de biologie/Centre de recherche en horticulture, Pavillon Alexandre-Vachon, Université Laval, Québec, QC, G1V 0A6, Canada

**Keywords:** Leaf proteome, Stress responses, Insect herbivory, Potato (*Solanum tuberosum* L.)

## Abstract

**Background:**

Various factors shape the response of plants to herbivorous insects, including wounding patterns, specific chemical effectors and feeding habits of the attacking herbivore. Here we performed a comparative proteomic analysis of the plant's response to wounding and herbivory, using as a model potato plants (*Solanum tuberosum* L.) subjected to mechanical wounding, defoliation by the Colorado potato beetle *Leptinotarsa decemlineata* Say, or phloem sap feeding by the potato aphid *Macrosiphum euphorbiae* Thomas.

**Results:**

Out of ~500 leaf proteins monitored by two-dimensional gel electrophoresis (2-DE), 31 were up- or downregulated by at least one stress treatment compared to healthy control plants. Of these proteins, 29 were regulated by beetle chewing, 8 by wounding and 8 by aphid feeding. Some proteins were up- or downregulated by two different treatments, while others showed diverging expression patterns in response to different treatments. A number of modulated proteins identified by mass spectrometry were typical defense proteins, including wound-inducible protease inhibitors and pathogenesis-related proteins. Proteins involved in photosynthesis were also modulated, notably by potato beetle feeding inducing a strong decrease of some photosystem I proteins. Quantitative RT PCR assays were performed with nucleotide primers for photosynthesis-related proteins to assess the impact of wounding and herbivory at the gene level. Whereas different, sometimes divergent, responses were observed at the proteome level in response to wounding and potato beetle feeding, downregulating effects were systematically observed for both treatments at the transcriptional level.

**Conclusions:**

These observations illustrate the differential impacts of wounding and insect herbivory on defense- and photosynthesis-related components of the potato leaf proteome, likely associated with the perception of distinct physical and chemical cues *in planta*.

## Background

Mechanical wounding has often been used in experimental setups to mimic insect herbivory, based on the well documented upregulation of several genes and proteins in wounded plants that are also upregulated by chewing herbivores [1]. It is now well established however that these stress cues induce a number of distinct responses in plants, owing to the complex physical and chemical interactions established between the herbivores and their host plant [1-5]. An early example of this was provided by Korth and Dixon [6], who reported a fast accumulation of mRNA transcripts for 3-hydroxy-3-methylglutaryl-coenzyme A reductase and wound-inducible proteinase inhibitor II (Pin-II) in potato leaves attacked by the lepidopteran pest *Manduca sexta*, compared to a slower accumulation in mechanically wounded leaves. Another early example was provided by Reymond et al. [7], who monitored the expression of ~150 defense-related genes in Arabidopsis leaves using DNA microarrays, and showed that many genes previously described as 'wound-inducible' were not upregulated upon feeding by caterpillars of the Small Cabbage White *Pieris rapae*.

Oral secretions introduced in wound tissues during insect feeding are known to play a central role in the observed responses [6,8-18]. Wounding patterns during herbivory also have an impact on the plant's responses [19,20], as well as specific interactions established between plants and herbivores of different feeding guilds [21-24]. Mithöfer et al. [19] showed for instance that sustained mechanical wounding applied to lima bean leaves in such a way as to reproduce the leaf removal pattern observed with *Spodoptora littoralis* larvae is required to reproduce volatile emission patterns similar to those induced by the insect. It was also documented that chewing herbivores, such as caterpillars, do not induce the same set of defense responses as piercing-sucking insects such as aphids, which obtain their nutrients directly from the phloem [21,22]. Aphids establish a prolonged interaction with their host plant, from which they take large quantities of phloem sap [25]. Their specialized mouthparts, or stylets, allow them to reach sieve tubes via an intercellular route, without causing major damage to plant tissues [26,27]. This feeding behaviour minimizing tissue injury translates into a unique type of plant-insect interaction, where defense genes induced *in planta* are in part similar to those induced by pathogenic infection [28,29].

All in all, data collected over the last several years illustrate the striking complexity of metabolic responses to biotic stress cues in plants, which obviously implicate the specific and coordinated regulation of several genes, proteins and metabolites. From an experimental viewpoint, non-biased 'omics' strategies involving transcriptomics, proteomics or metabolomics are of particular value for deciphering complex stress-related processes in plant systems [30-33]. For instance, classical proteomic approaches involving two-dimensional gel electrophoresis (2-DE) and mass spectrometry (MS) [34] are well suited to simultaneously monitor the hundreds of proteins characterizing plant-arthropod interactions (e.g. [35-40]). In the present study, we used a 2-DE/MS approach to compare the response of cultivated potato *Solanum tuberosum* to either mechanical wounding or herbivory by two specialized insect herbivores, the defoliating pest Colorado potato beetle *Leptinotarsa decemlineata* Say and the potato aphid *Macrosiphum euphorbiae* Thomas. Previous studies reported specific metabolic effects for the regurgitant of Colorado potato beetles in leaves of Solanaceae. Kruzmane et al. [41] reported a significant increase of ethylene biosynthesis, peroxidase activity and polyphenol oxidase activity in wounded potato leaves treated with this fluid. In a transcriptomic study with EST microarrays for Solanaceae species, Lawrence et al. [42] reported the induction of 73 genes in wounded potato leaves treated with potato beetle regurgitant, concomitant with the repression of 54 other genes. An interesting example of gene repression mediated by the oral secretions of potato beetle was provided by the same group [43], who showed the ability of a [10,30]-kDa fraction of the regurgitant to inhibit the expression of two wound-inducible, defense-related proteinase inhibitor genes in tomato leaves. Here we report differential effects for mechanical wounding, beetle leaf chewing and aphid phloem sap feeding on the steady-state levels of defense- and photosynthesis-related proteins in potato leaves.

## Results

### Differential gene-inducing effects among treatments

**Figure 1:**
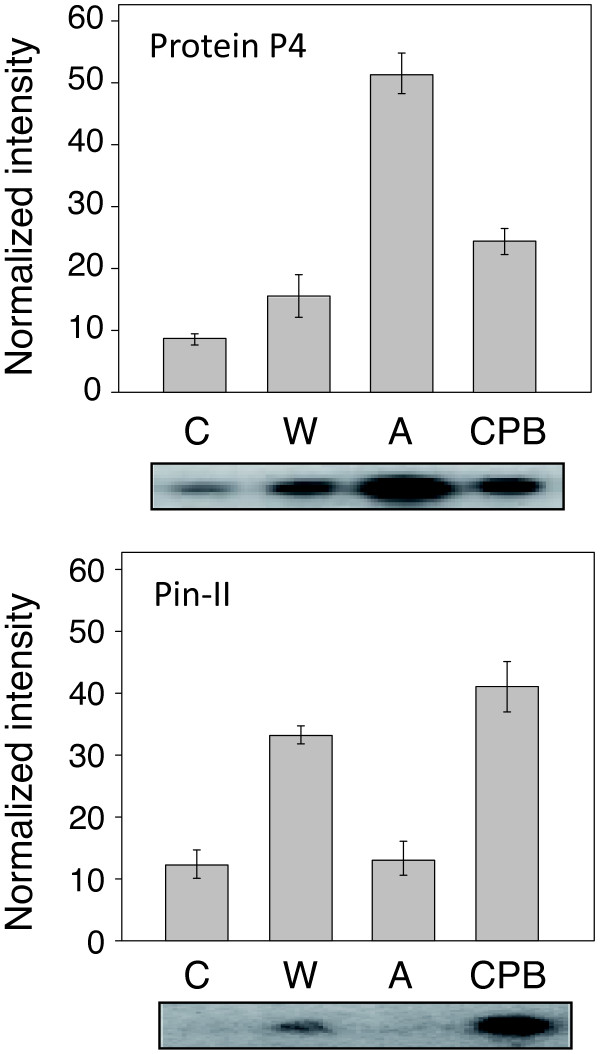
**Northern blot analysis for the induction of proteinase inhibitor II (Pin-II) and PR protein P4 mRNA transcripts by wounding (W), potato aphid feeding (A) or Colorado potato beetle chewing (CPB) in potato leaves.** Each bar is the mean of three independent (plant replicate) values ± se. Leaf total RNA was extracted 24 h after initiating the stress treatments. The membranes were hybridized with ^32^P-labelled cDNA probes for either Pin-II or Protein P4. Equal RNA loading in each well was controlled by ethidium bromide fluorescence of total RNA fixed onto the membrane. C, control leaves from healthy, non-treated plants.

A northern blot analysis was first carried out with probes for the mRNA transcripts of Pin-II and pathogenesis-related (PR) protein P4 to confirm the gene inducing effects of wounding, potato beetle chewing and potato aphid phloem sap feeding (Figure 1). Previous studies described the differential effects of chewing insects, sap-feeding insects, the wound hormone jasmonic acid and a number of pathogen-derived elicitors on the induction of Pin-II and protein P4 in leaves of Solanaceae (e.g. [44-47]). As expected, mRNA transcripts for Pin-II, a wound-inducible protein, were easily detected in leaf extracts of plants subjected to wounding or potato beetle chewing while remaining at lower levels in control and aphid-treated plants (Student's *t*-test; *P* = 0.0001). By contrast, mRNA transcripts for protein P4, a pathogen-inducible protein also induced by aphids [45], were detected, respectively, at very high and moderate levels in aphid- and potato beetle-treated plants, compared to lower basic levels in control and mechanically wounded plants (Student's *t*-test; *P* < 0.0001). These observations confirming distinct gene inducing effects among treatments were also suggesting the occurrence of distinct protein complements in potato leaves upon wounding or challenge with biotic stress agents.

### Control, wounded and insect-treated leaves exhibit distinct proteome patterns

**Figure 2:**
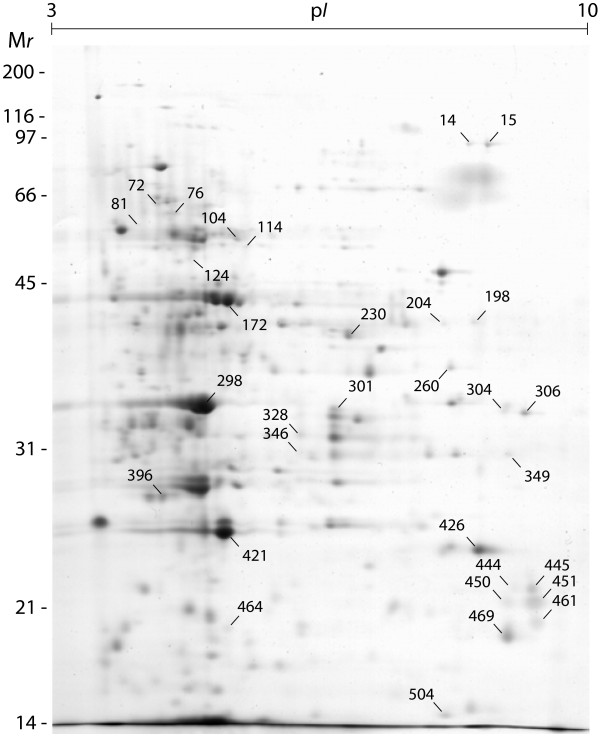
**Gel image for the proteome of potato leaves submitted to potato aphid phloem sap feeding, as visualized after Coomassie blue staining following 2-DE.** Protein spot numbers point to proteins up- or downregulated following mechanical wounding, Colorado potato beetle leaf chewing or potato aphid feeding. A non-linear 3 to 10 p*I* gradient was used for IEF. M*r* values on the left refer to commercial molecular weight protein markers (kDa). A total of 167 μg of protein was loaded on each 2-D gel. Proteins synthesized *de novo* upon wounding or potato beetle chewing are not shown on this gel.

**Figure 3:**
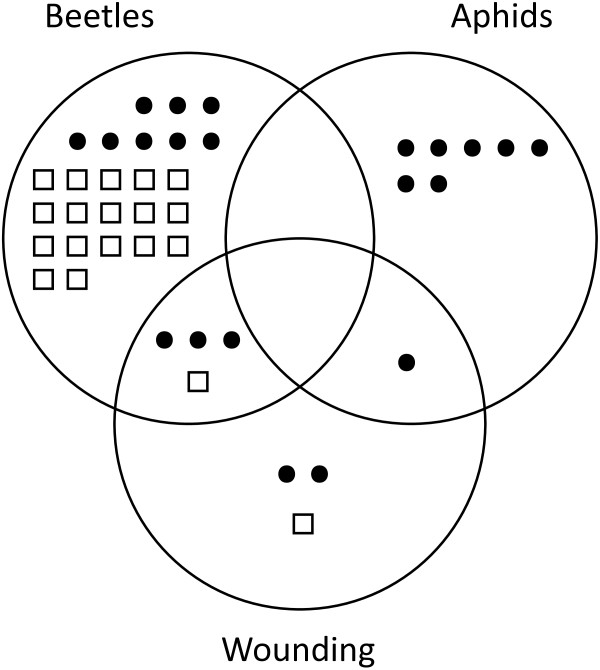
**Venn diagram for the relative number of proteins specifically or co-regulated in potato leaves following mechanical wounding, potato beetle chewing or potato aphid sap feeding.** Black circles represent upregulated proteins, blank squares downregulated proteins. Any significantly regulated protein is represented more than once if showing diverging expression trends for different treatments.

A comparative proteomic study involving image analysis following 2-DE was conducted to test this hypothesis, using leaf protein extracts from control, wounded and insect-treated plants. The abundant protein ribulose-1,5-bisphosphate carboxylase/oxygenase (rubisco) was barely detectable in 2-D gels, as reported earlier for potato leaf proteins extracted under similar acidic conditions [48,49]. Of more than 500 proteins detected (Figure 2), 31 were up- or downregulated by at least twofold in treated plants compared to their basic level in untreated control plants (anova; *P* < 0.05), including three proteins produced *de novo* following wounding and/or potato beetle feeding (Table 1). The relative number of up- and downregulated proteins in leaves, and the expression trend of each modulated protein compared to control plants, differed depending on the stress exerted. Potato beetle feeding had, by far, the strongest impact, with 29 proteins modulated in leaf extracts, compared to 8 proteins for both wounding and the aphid treatment (Table 1, Figure 3). Most proteins modulated by mechanical wounding or aphid feeding were upregulated compared to the control, in sharp contrast with the downregulation of 18 proteins, out of 29 modulated, by potato beetle feeding (Table 1). None of the proteins modulated by at least one treatment was affected by all stress treatments, indicating a strong specificity of the plant's stress response at the proteome scale.

**Table 1:** **Relative levels of potato leaf protein spots exhibiting a more than twofold decrease or increase for at least one stress** treatment (see Figure 2 for protein spot numbering) ^***a***^

		**Stress **^***b***^		
**Spot**	**Protein (NCBI Accession No.) **^***c***^	**Wounding**	**Beetles**	**Aphids**
14		1.3	0	4.6
15	EST537399 similar to a subtilase (NP_199378)	1.6	0	4.1
72	Rubisco subunit binding-protein alpha subunit (P08824)	1.9	4.1	1.0
76	EST396117 similar to protein disulfide isomerase (Q9XF61)	0.6	3.2	1.0
81		0.4	8.5	0.8
104	ATP synthase β subunit (AAM52206)	1.3	0.4	3.5
114		0.5	0	1.0
124		1.1	2.5	1.0
172 ^*d*^	Rubisco activase (AAC15236)	0.7	0.9	1.3
198		1.1	0.3	0.8
204		0.8	0	0.6
230		0.8	0.3	1.3
260		0.9	0	0.8
298 ^*d*^	33-kDa PSII oxygen evolving complex protein (CAA35601)	0.9	0.9	1.0
301		1.3	0.4	0.9
304	Rubisco large subunit fragment (CAA70392)	0.9	0	1.0
306		1.2	0	1.5
328		2.5	2.7	0.9
346		2.5	2.3	0.7
349	Putative l-ascorbate peroxidase, chloroplastic (Q9THX6)	1.1	0.2	1.4
396	Cysteine protease inhibitor 7 (O24385)	1.7	2.6	0.8
421 ^*d*^	23-kDa PSII oxygen evolving protein (CAA67696)	0.9	1.0	1.1
426	Aspartic protein inhibitor 3 (P58518)	2.0	0.6	1.0
444	Photosystem I reaction center subunit II (P12372)	2.5	0	1.2
445		2.3	0	2.4
450		1.8	0	2.4
451	Photosystem I reaction center subunit IV B (Q41229)	1.5	0	2.4
461	Photosystem I reaction center subunit IV (P12354)	1.3	0	4.3
464	Aspartic protease inhibitor (CAA45723)	1.3	2.9	1.4
469	Aspartic protein inhibitor 3 (P58518)	1.0	0	1.0
504	Pathogenesis-related protein P2	1.5	1.3	4.1
526			N (0.029)	
527		N (0.011)	N (0.056)	
528			N (0.089)	

^*a*^Statistical significance of the observed variations was confirmed by a one-way analysis of variance, using a significance threshold (α value) of 0.05.

^*b*^Data refer to average normalized volumes of the spots, divided by the average normalized volume for the corresponding spot in control gels. N stands for proteins induced *de novo* following stress treatment. Numbers in parentheses indicate averaged normalized volumes.

^*c*^Information on MS data, protein identifications and 2-DE coordinates is given in Additional files 1 and 2.

^*d*^Unmodulated, negative controls found at comparable levels in treated and control plants.

A detailed assessment of the plant's responses to wounding and potato beetle feeding was undertaken to further confirm the differential effects of these treatments in leaves (Table 2). In theory with a 3-state response (unregulated, upregulated, or downregulated), nine (i.e. 3^2^) different scenarios may describe the effects of two independent treatments on the expression of a single gene or protein, including no response to either treatment (1 scenario); up- or downregulation by only one treatment (see Table 2, 4 scenarios); up- or downregulation by both treatments (2 scenarios); and contrasting effects causing an up- (or down-) regulation effect by one treatment, concomitant with a down- (or up-) regulation by the other treatment (2 scenarios). In the present case, at least one modulated protein was detected for seven, out of eight, possible response scenarios (Table 2). A majority of proteins were modulated by only one treatment (23 proteins, out of 30 proteins modulated, overall), with specific downregulation by potato beetle chewing representing the most common situation (15 proteins). Interestingly, some proteins were up- or downregulated by both wounding and potato beetle feeding (Protein spots 114, 328, 346 and 527), while some others showed diverging, contrasting patterns strongly suggesting specific effects *in planta* (Protein spots 81, 444 and 445). A similar conclusion could be drawn for the beetle and aphid treatments, where most proteins modulated by aphid feeding were also modulated by beetle chewing, albeit in a contrasting manner (see Table 1).

**Table 2:** **Possible expression scenarios (excluding no response to either treatment) for stress-regulated proteins in potato leaves submitted to mechanical wounding or potato beetle chewing**^*a*^

**Scenario**	**Wounding**	**Potato beetles**	**Regulated proteins**
1	↑	-	426
2	-	↑	72, 76, 124, 396, 464, 526, 528
3	↓	-	none
4	-	↓	14, 15, 104, 198, 204, 230, 260, 301, 304, 306, 349, 450, 451, 461, 469
5	↑	↑	328, 346, 527
6	↓	↓	114
7	↑	↓	444, 445
8	↓	↑	81

^*a*^Regulated protein spots correspond to those proteins showing a more than twofold significant increase or decrease in wounded or potato beetle-treated leaves, compared to healthy control leaves (see Table 1). Upward arrows indicate an upregulating effect following wounding and/or potato beetle feeding; downward arrows indicate a downregulating effect.

### Potato beetles and aphids differentially impact photosynthesis-related proteins

**Figure 4:**
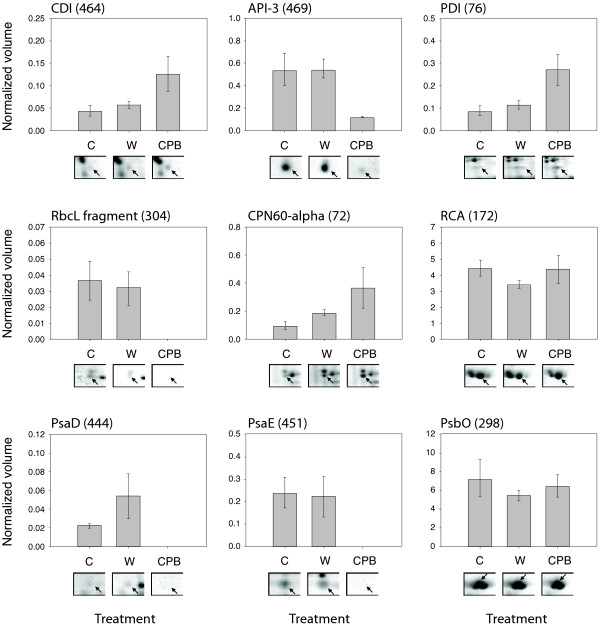
**Selected defense- and photosynthesis-related proteins identified as modulated in potato leaves submitted to wounding (W) or Colorado potato beetle (CPB) feeding.** Bars represent average normalized volumes in 2-D gels ± se; gel samples illustrate protein level alterations after 24 h. Numbers in parentheses refer to protein spot numbers on 2-D gels (see Figure 2). C, control, 'no-stress' treatment; CDI, cathepsin D inhibitor; API-3, aspartic protease inhibitor 3; PDI, protein disulfide isomerase; RbcL fragment, fragment of rubisco large subunit; CPN60-alpha, rubisco subunit-binding protein alpha subunit; RCA, rubisco activase; PsaD, photosystem I reaction center subunit II; PsaE-B, photosystem I reaction center subunit IV B; PsbO, 33-kDa photosystem II oxygen evolving complex protein.

**Figure 5:**
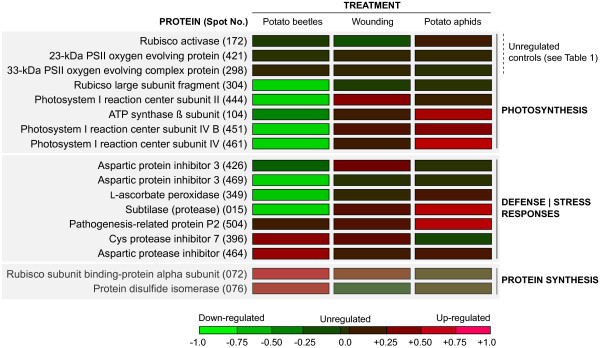
**'Heat map' for proteins up- or downregulated in potato leaves submitted to wounding, potato beetle chewing or aphid sap feeding.** Data are presented as log values of relative increases (in red) or decreases (in green) compared to control (untreated) leaves (zero value). Modulated proteins could be categorized in three functional groups, namely photosynthesis-related proteins, defense-related proteins, and proteins involved in protein biosynthesis and maturation. More than tenfold increases (or decreases) were assigned an arbitrary value of +1.0 (or −1.0). The map was generated using the 'Lab' tool of Adobe Illustrator CS3, v. 13.0.0 (Adobe Systems, San Jose CA, USA).

Several proteins exhibiting an altered content in wounded or insect-treated leaves were confidently identified by MALDI TOF MS or ion trap MS/MS (Figure 4, Table 1, Additional file 1 and Additional file 2). As expected, a number of these proteins corresponded to well-characterized stress-related inducible proteins, including Asp and Cys protease inhibitors, PR protein P2, and chloroplastic l-ascorbate peroxidase. Proteins constituent of the photosynthetic apparatus or functionally involved in photosynthesis were also identified, in line with the reported impact of insect herbivory on this physiological process (e.g. [50-52]). An ATP synthase β protein subunit and a number of photosystem I protein components were downregulated in response to potato beetle feeding, in contrast with wounding and aphid feeding having no impact, or an upregulating impact, on these proteins (Figure 5).

**Figure 6:**
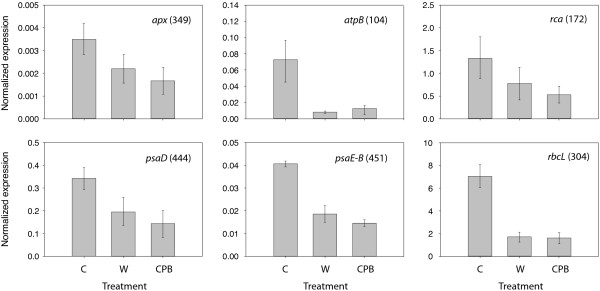
**mRNA transcripts for photosynthesis-related proteins in potato leaves submitted to wounding (W) or Colorado potato beetle (CPB) feeding.** The transcripts were extracted after 24 h, and quantified by real-time RT PCR using appropriate oligonucleotide primers (see Table 3 for details). Each bar is the mean of three independent (plant replicate) values ± se. Numbers in parentheses refer to protein spot numbers on 2-D gels (see Figure 2). C, control healthy plants.

Real-time reverse transcriptase (RT) PCR assays were conducted with nucleotide primers for different photosynthesis-related proteins (see Table 3) to compare the impact of wounding and potato beetle feeding at the transcriptome level (Figure 6). Preliminary tests with primers for Pin-II and protein P4-encoding genes (not shown) and the above-described northern blot signals as a reference (see Figure 1) first allowed to validate the RT PCR amplifications under our experimental conditions. Interestingly, both wounding and beetle feeding induced a systematic downregulation of the genes monitored (Figure 6), including genes, such as *psaE-B* for photosystem I subunit IV-B (Protein spot 451) and *atpB* for ATPase subunit β (Spot 104), encoding proteins unaffected by wounding (see Table 1 and Figure 5). Transcription of the 'control' gene *rca*, shown on 2-D gels to encode a protein unaffected by the stress treatments (see Figure 4, Spot 172), was also downregulated.

**Table 3:** Oligonucleotide primers for real-time RT PCR

**Spot**	**Protein**	**Gene**	**Accession No.**^***a***^	**Forward primer (5’–3’)**	**Reverse primer (5’–3’)**	**Size (bp)**
104	ATPase β subunit	*atpB*	AY300043	ATGAGAGTTGGTTTGACTGC	CGAATTGTTTCTGCTAGACC	629
304	Rubisco (large subunit)	*rbcL*	M76402	GAACGTGAACTCACAACCAT	GACATACGTAACGCTTTTGC	351
172	Rubisco activase	*rca*	SGN-U243405	AATACACCGTCAACAACCAG	CACCAATGTTTTCAATTCCA	382
349	l-Ascorbate peroxidase (chloroplastic)	*apx*	SGN-U247328 + SGN-U258329	ATGAGGATCGCTTTCATAGAC	ATTTTCTGGTCTGCTGATCTC	311
444	PsaD (PSI subunit II)	*psaD*	STU556864	TGGAAACAATCCCTCCTATC	ACAAATTGGGTCCTTCTCTC	310
451	PsaE-B (PSI subunit IV-B)	*psaE-B*	SGN-U245041	CCTAATGTCACCTCTAACTCTG	TAAAACATGGAAAGCACAGG	458
---	Proteinase inhibitor II	*pinII*	L37519	AATCTTGGGTTTGGGATATG	TATGTGGATCGCAATTTAGG	178
---	PR-1 protein P4	*p4*	AJ250136	GCACAAAATTATGCCAACTC	AGTTGCATGAAATGAACCAC	271
---	Elongation factor 1-a	*ef1-α*	AB061263	ATTGGAAACGGATATGCTCCA	TCCTTACCTGAACGCCTGTCA	101

^*a*^Accession numbers from the NCBI GenBank Database ( http://www.ncbi.nlm.nih.gov) or the Solanaceae Genomics Network Database ('SGN' sequences; http://www.sgn.cornell.edu).

## Discussion

Previous studies documented the differential effects of wounding and insect feeding on the primary and defense metabolism of higher plants, based on the monitoring of model genes and proteins, or, more recently, on the use of 'omics' approaches for a systemic analysis of mRNA transcript or protein complements. Transcriptomics has been instrumental over the years to decipher complex physiological processes in plant-insect systems, which often implicate dynamic cross-talks between defense pathways and the regulation of numerous genes in host plant tissues (e.g. [21,22,42,53-56]). Studies also confirmed the usefulness of proteomics in recent years, for the elucidation of plant-insect interactions at the proteome and metabolic levels (e.g. [35-40]). Our data pointing to the onset of distinct protein regulation patterns in mechanically wounded and insect-treated plants despite similar transcriptional control patterns here illustrate the often described discrepency between transcriptomic and proteomic data generated from complex biological systems, including plant-insect systems [39]. From a biological standpoint, they may reflect in part the regulatory role of metabolic effectors in the oral secretions of attacking herbivores, and confirm the relevance of proteins as useful biomarkers for a realistic account of the situation in vivo.

About thirty proteins had their concentration increased or decreased by at least twofold in wounded or potato beetle-treated potato leaves under our experimental conditions, similar to Giri et al. [35] reporting the modulation of 18 proteins, out of approximately 500 monitored, in leaves of *N. attenuata* challenged with *M. sexta* larvae. Most interestingly, none of the proteins modulated here was up- or downregulated by all three stress treatments, and most of them were up- or downregulated by only one treatment (see Figure 3). These observations, together with the unaltered normalized volumes observed on 2-D gels for more than 90% of the proteins detected, underline the remarkable stability of the host plant's leaf proteome under various stress conditions. They also underline, in accordance with the treatment-specific expression patterns observed for Pin-II and protein P4 mRNA transcripts (Figure 1), the onset of stress-specific responses *in planta*. The differential, even diverging expression patterns observed for a number of proteins following different stress treatments (Table 2) highlight, in particular, the diversity of possible responses, not only involving the identity and expression rate of several genes and proteins, but also their accumulation trend in plant tissues.

Experimental biases influencing data interpretation, such as the focus on abundant proteins during 2-DE, the adoption of a conservative twofold threshold for protein spot selection or the use of a wound treatment not exactly reproducing the injury pattern observed during insect feeding, cannot be excluded *de facto*. It is well known that leaf damage pattern, intensity and duration may significantly impact stress perception and wound hormone (e.g. jasmonic acid) accumulation in wounded plants, with a likely impact on stress metabolic pathways and defense gene inductions [20,57]. Nevertheless, a number of observations support the hypothesis of distinct effects for the three treatments assessed. For instance, the limited and specific effects of potato aphids could be expected *a priori* given the feeding habits of these insects and their limited impact on the structural integrity of host leaf tissues [27]. Proteomic data indicating the upregulation of a PR-4 protein (Spot 504) upon aphid feeding was also in line with previous reports on plant-aphid interactions and those models proposing different recognition schemes *in planta* in response to phloem sap feeding and chewing arthropods [28,45,58,59].

Interpretation issues may remain more problematic for the mechanical wound treatment, but a number of observations, such as the detection of an Asp protease inhibitor isoform that is upregulated exclusively in wounded leaves (Spot 426) and the diverging accumulation trends of photosystem I reaction center subunit II (Spot 444) in leaves subjected to wounding and potato beetle feeding, indeed suggest differential, treatment-specific effects. Most convincingly, RT PCR data showed comparable repressing effects for wounding and potato beetle treatments on the transcription of some photosynthesis-related genes, despite clearly divergent accumulation trends on 2-D gels for the corresponding proteins (Spots 104, 304, 444 and 451). These findings suggest overall the onset of stress-specific gene and protein control mechanisms in the host plant involving a combination of regulatory events common to different stress cues, and treatment-specific regulatory events leading to distinct responses at the proteome level. In the present case, the differential metabolic effects of wounding and potato beetles could have been the result of common transcriptional regulation events triggered by wounding and the 'wound hormone' jasmonic acid [60], combined with specific effects of the potato beetle regurgitant [42,43] post-translationally altering the turnover of some regulated proteins *in planta*.

In line with an earlier study reporting the induction of several defense-related genes in potato leaves challenged with potato beetle larvae [61], defense-related proteins such as PR-proteins (e.g. proteins P2 and P4) and wound-inducible protease inhibitors were upregulated in leaves by wounding, potato aphid and/or potato beetle treatments. Of interest was, by contrast, the downregulation of a Kunitz Asp protease inhibitor in potato beetle-treated plants (Spot 469) (Figures 4 and 5). Asp protease inhibitors are well-characterized wound-/jasmonate-inducible proteins in potato leaves [62], known to inhibit digestive Asp proteases in the Colorado potato beetle larval midgut [63,64]. From an ecological viewpoint, the downregulation of an Asp protease inhibitor and the slight repression of a second isoform despite a twofold upregulation in wounded leaves (Spot 426) (Figure 5) could represent an advantage for the insect in vivo. The repression of Ser protease inhibitor-encoding genes in wounded tomato leaves treated with potato beetle regurgitant has been reported earlier [43], as well as the repression of two trypsin inhibitor-encoding genes in Arabidopsis leaves by oral secretions of the lepidopteran herbivore *Spodoptera littoralis*[56]. The biological significance of protease inhibitor downregulation in potato leaves remains equivocal in the present case given the number and diversity of protease inhibitor isoform- (including Kunitz inhibitor isoform-) encoding genes in the potato genome [65], but it is tempting to speculate about a possible evasive strategy of the insect to elude detrimental digestive protease inhibition. In a similar way, the downregulation of an ATP synthase β subunit (Spot 104) (Figure 5) in potato leaves could contribute to attenuate the impact of host plant defenses and help maintain the insect's fitness, given the role attributed to host plant ATP synthase fragments as "non-self" triggers of defense responses upon herbivory [14].

The downregulation of photosynthesis-related proteins in potato beetle-treated plants is more difficult to interpret. Proteomic and transcriptomic studies have already documented the post-translational modification [66] or the downregulation of rubisco, rubisco activase or other photosynthesis-related proteins in insect-challenged leaves or in wounded leaves treated with insect oral secretions [35,39,67]. Several hypotheses have been proposed to explain these observations in terms of metabolic strategies to sustain plant's or herbivore's fitness. A number of authors have suggested the need for a reallocation of carbon resources towards defense responses in the host plant [67-69]. The degradation of rubisco, in this perspective, would provide the plant with an important source of amino acids for newly synthesized defense proteins [52,70], while also lowering the nutritive value of leaf tissues by limiting dietary protein availability to the aggressor [71].

Two alternative, although non-exclusive hypotheses could explain the observed effects: (1) the secretion of biochemical effectors in the insect regurgitant which might limit energy resources available to the host plant or promote the accumulation of compounds, such as reactive oxygen species, toxic to plant cells [72,73]; and (2) a general disturbance of the whole plant system under stress conditions which might negatively affect photosynthesis and induce compensatory responses. The strong and specific downregulation of chloroplastic l-ascorbate peroxidase (Spot 349) and photosystem I proteins (Spots 444, 451 and 461) observed here following potato beetle feeding (Figure 5) is compatible with the hypothesis of reduced energy production and increased accumulation of toxic oxidizing molecules. Specific upregulation of the 60-kDa chaperonin α-subunit rubisco chaperone (Spot 72) and protein disulfide isomerase (Spot 76) upon potato beetle treatment, along with the maintenance of rubisco activase (Spot 172) content, support the idea of compensatory responses to sustain protein biosynthesis, folding and assembly. Overexpression of the 60-kDa rubisco chaperone, also observed in tobacco leaves attacked by *M. sexta* larvae [74], could represent a general strategy for the plant to preserve rubisco assembly under stress conditions. Work is underway to assess the net impacts of mechanical wounding and potato beetle herbivory on photosynthesis in potato leaves, keeping in mind the striking plasticity of major physiological processes in plants. Work is also underway to further dissect the inducing effects of the potato-potato beetle system components, including the plant itself. Studies have described in recent years the elicitor activity of plant-derived compounds in plant-herbivore systems, including plant protein fragments coming back to the host plant via insect regurgitant [14,15,75-77].

## Methods

### Plants

Thirty-five days-old potato plants (*Solanum tuberosum* L., cv. Superior) cultivated in a Conviron growth chamber (Conviron, Winnipeg MB, Canada) were used for the experiments. The plants were grown in 2-gallon pots in Promix BX substrate (Premier Tech Horticulture, Rivière-du-Loup QC, Canada), watered as needed and fertilized once a week with a 200 ppm solution of 20–20–20 adjusted to pH 5.8 with H_3_PO_4_. Environmental conditions in the growth chamber were maintained as follows: a light intensity of 125 μEinstein m^-2^.s^-1^, a 14/10 h L:D photoperiod, a 24°C /18°C L:D thermoperiod, and a relative humidity of 60%.

### Stress treatments

The study involved four treatments: (i) artificial wounding with a razor blade; (ii) defoliation with 4^th^-instar Colorado potato beetle larvae (*L. decemlineata* Say); (iii) sap feeding with 2^nd^-instar potato aphids (*M. euphorbiae* Thomas); and (iv) a 'no-stress' control treatment with unchallenged, healthy plants. The wounding treatment consisted of three 1 cm-long cuts through the leaf lamina with a sterile razor blade on a terminal leaflet of the plant's 4^th^ upper leaf, followed by the same treatment 2 h later on another terminal leaflet, then repeated after 4 h on the 3^rd^ terminal leaflets. For the potato beetle treatment, one 4^th^-instar larva reared on potato plants (cv. Superior) was placed for 24 h onto the adaxial side of the 4^th^ upper leaf, in such a way as to leave about 50% of the initial leaf surface at the end of the treatment. For the sap feeding treatment, 50 aphids reared on potato plants (cv. Superior) were deposited onto the adaxial side of the 4^th^ upper leaf and left to feed for 24 h. All insects were confined to the treated leaves using specially designed cages made of clear plastic and nylon. Each treatment included three repetitions, with plant replicates distributed randomly in the growth chamber. Cages for insect confinement were fixed on all plants, including wounded (leaflet-cut) and control plants, to avoid confounding effects due to the experimental setup. The 4^th^ (treated) and 3^rd^ (younger) upper leaves of treated and control plants were collected and pooled after 24 h for each plant replicate, ground to a fine powder in liquid nitrogen, and kept at −80°C until further analysis.

### Northern blotting

mRNA transcripts for Pin-II- and protein P4 were visualized by northern blotting as described earlier [46], with total RNA extracted from potato leaves according to Logemann et al. [78]. The probe for protein P4 was amplified by PCR from a leaf RNA population of benzothiadiazole–treated tomato plants (*Solanum lycopersicum*), using the following oligonucleotide primers (GenBank Accession Number M69247): [5’–AAATGGGGTTGTTCAACATCTCATTG–3’]/[5’–CAATAATAATAGGATATCAATCCGATCCAC–3’]. The probe for Pin-II was amplified from methyl jasmonate-treated tomato leaves using the following primers (Accession Number K03291): [5’–GCCAAGGCTTGTACTAGAGAATGTGGT–3’]/[5’–GGACAAGTCTAGAGTCACATTACAGGGTAC–3’]. For northern blot analysis, 10 μg of total RNA was resolved in 1.2% (w/v) formaldehyde-agarose gels and blotted onto nitrocellulose membranes [78]. The membranes were hybridized for 20 h with ^32^P-labelled DNA probes and washed under stringent conditions. The filters were subjected to autoradiography for 24 h at −80°C, using intensifying screens. All assays involved three independent (plant) replicates, to allow for statistical assessment of the data.

### Real-time RT PCR

mRNA transcripts for Pin-II, protein P4 and different photosynthesis-related proteins were quantified by real-time RT PCR as described earlier [79], using a Roche LightCycler apparatus [System 1.0] and the LightCycler-FastStart DNA Master SyBRGreen I kit (Roche Diagnostics, Laval QC, Canada). Leaf total RNA was extracted with the Plant RNA Reagent (Life Technologies, Burlington ON, Canada) following the supplier's instructions, and contaminant DNA was removed by treatment with DNase I (Roche Diagnostics). First-strand cDNA was produced with 2 μg of total RNA using the Qiagen’s Omniscript RT kit (Qiagen, Mississauga ON, Canada). Two hundred ng of reverse-transcribed RNA was used for amplification with specific oligonucleotide primers (Table 3). The specificity of RT PCR product formation was confirmed by melting curve analysis and gel electrophoresis. Elongation factor 1-α was used as a control (housekeeping) gene for the tests [80]. All assays involved three independent (plant) replicates, to allow for statistical assessment of the data.

### Sample preparation for 2-DE

Leaf proteins for 2-DE were extracted from frozen leaf powder (see above), essentially as described by Damerval et al. [81]. In brief, 300 μg of leaf powder was precipitated in 1 mL of 10% (v/v) trichloroacetic acid/0.07% 2-mercaptoethanol diluted in acetone, for 2 h at −20°C. After centrifugation at 20,000 *g* for 25 min at 4°C, the pellets were washed four times with 1 mL of acetone containing 0.07% (v/v) 2-mercaptoethanol. The pellets were vacuum-dried in a SPD121 Thermo Savant SpeedVac centrifuge (Thermo Fisher Scientific, Mississauga ON, Canada) for 15 min at 20°C, and the proteins resolubilized by sonication for 1 h at 30°C, in 60 μL of electrophoretic sample buffer [8 M urea containing 2% (v/v) CHAPS, 0.5% (v/v) IPG buffer 3–10 (GE Healthcare, Baie d'Urfé QC, Canada) and 60 mM dithiothreitol] per mg of dried pellet. Protein concentration in the extracts was determined according to Ramagli and Rodriguez [82], with ovalbumin as a standard.

### 2-DE

2-DE first involved isoelectric focusing (IEF) (1^st^ dimension), followed by 12% (w/v) SDS-PAGE (2^nd^ dimension) [83]. IEF was performed in 13-cm Immobiline DryStrip gel strips (GE Healthcare) along a 3 to 10 pI gradient, with 167 μg of leaf protein per gel strip. Proteins were applied on the strips and resolved using an IPGphor apparatus (GE Healthcare). The program for IEF involved the following sequential steps: rehydration at 30 V for 12 h; 100 V for 1 h; 500 V for 1 h; 1,000 V for 1 h; 5,000 V for 1 h; and 8,000 V to reach 25,960 Vh. Following IEF, the strips were incubated twice for 20 min in Tris–HCl equilibration buffer, pH 8.8, containing 6 M urea, 30% (v/v) glycerol, 2% (w/v) SDS and 0.1% (v/v) dithiothreitol (or 5% (v/v) iodoacetamide for the second incubation), and used immediately for the second dimension. SDS-PAGE [84] was performed at 200 V in 1-mm thick polyacrylamide slab gels, using a PROTEAN Plus Dodeca Cell unit (Bio-Rad, Mississauga ON, Canada) allowing for the simultaneous processing of twelve gels. After migration, the gels were fixed overnight in water containing 10% (v/v) acetic acid and 50% (v/v) methanol. The proteins were stained with the Bio-Safe Coomassie Blue reagent (Bio-Rad), following the supplier's instructions.

### Gel image analysis

Image analysis was carried out as described earlier [48] using the Phoretix 2-D Expression software, v2005 (NonLinear USA Inc, Durham NC, USA), after digitalizing the gels with an Amersham Image Scanner digitalizer and the ImageMaster LabScan software, v3.0 (GE Healthcare). Automatic spot detection and ‘non-spot background’ subtraction were performed following the supplier’s instructions to eliminate staining background inherent to the image capture process. The gel containing the highest number of protein spots was identified, and used as a reference gel for protein spot matching. An average virtual gel was constructed for each set of three gels (three biological replicates), which included protein spots found on at least two gels. Average spot intensities were normalized to the total spot volume with a multiplication factor of 100 to minimize errors due to differences in staining intensity or in the amount of protein loaded. Spot matching was performed with the average gels, and those spots showing more than twofold differences in density were selected for protein identification. Statistical significance of the observed variations was confirmed by a one-way analysis of variance, using a significance threshold (α value) of 0.05.

### Mass spectrometry analyses

Protein spots for identification were excised manually from the gels, digested with sequencing grade trypsin (Promega, Madison WI, USA), and sent to the Québec Genomics Center's Proteomics platform (Centre de recherche du CHUL, Québec QC, Canada) for matrix-assisted laser desorption ionization time-of-flight mass spectrometry (MALDI-TOF MS) or ion trap MS/MS analysis. In-gel protein digestion was performed on a MassPrep liquid handling station (Waters, Lachine QC, Canada), according to the manufacturer's specifications. The peptides were lyophilized and resuspended in 3 μL of 0.1% (v/v) trifluoroacetic acid in water until further analysis. The matrix used for MALDI-TOF MS was α-cyano-4-hydroxycinnamic acid diluted at 20 mg.mL^-1^ in 50% (v/v) acetonitrile/0.1% (v/v) trifluoroacetic acid. Equal volumes of peptides and matrix solutions were mixed, and 1 μL of the resulting mixture was spotted on a stainless steel MALDI sample plate. The solution was allowed to air-dry at 20°C, and washed three times with 2 μL of 0.1% (v/v) trifluoroacetic acid. MALDI-TOF MS spectra were acquired on a Voyager-DE PRO Biospectrometry Workstation (Applied Biosystems) in the positive-ion reflector delayed-extraction mode, and analyzed using the DataExplorer software, v4.0 (Applied Biosystems, Streetsville ON, Canada). MS/MS peptide spectra were generated by microcapillary reverse-phase chromatography coupled to an LCQ DecaXP (Thermo Fisher Scientific) quadrupole ion trap mass spectrometer with a nanospray interface. A 10-μL aliquot of the peptide sample was loaded onto a 75-μm internal diameter C18 picofrit column (New Objective, Woburn MA, USA). The peptides were eluted along a water-acetonitrile/0.1% (v/v) formic acid gradient, at a flow rate of 200 nL.min^-1^.

### Protein identification

MALDI-TOF MS spectra were analyzed using the Rockefeller University ProFound algorithm for protein identification, v4.10.5 ( http://prowl.rockefeller.edu/cgi-bin/ProFound), with the following search criteria: a maximum of one missed trypsin cleavage, complete carboxyamidomethylation of cysteine residues, methionine residues in the oxidized form, and maximal mass deviation of 100 ppm. MS/MS spectra were analyzed using the SEQUEST [85] and MASCOT [86] algorithms, with an MS/MS deviation tolerance of 0.5 Da and a peptide deviation tolerance of 2 Da. All MS data were searched against non-redundant Viridiplantae entries of the National Center for Biotechnology Information database ( http://www.ncbi.nlm.nih.gov).

## Abbreviations

2-DE: Two-dimensional gel electrophoresis; IEF: Isoelectic focusing; MALDI TOF MS: Matrix-assisted laser desorption ionization time-of-flight mass spectrometry; Pin-II: Potato proteinase inhibitor II; PR protein: Pathogenesis-related protein; RT: Reverse transcriptase; Rubisco: Ribulose-1,5-bis-phosphate carboxylase oxygenase; SDS-PAGE: Sodium dodecyl sulfate polyacrylamide gel electrophoresis.

## Competing interests

The authors declare that they have no competing interests.

## Authors' contributions

MOD contributed to the experimental design, performed the experiments, and wrote a first draft of the manuscript. CC contributed to the experimental design and writing of the manuscript. DM conceived the study, contributed to the experimental design, coordinated the experiments, and prepared the last version of the manuscript. All authors read and approved the final manuscript.

## Supplementary Material

Additional file 1 MALDI-TOF MS identification of potato leaf proteins regulated by wounding, potato beetle feeding or aphid phloem sap feeding. Click here for file

Additional file 2 Ion trap MS/MS identification of potato leaf proteins regulated by wounding, potato beetle feeding or aphid phloem sap feeding. Click here for file
