# Supplementary material for: Wounding, insect chewing and phloem sap feeding differentially alter the leaf proteome of potato, Solanum tuberosum L
Source: Proteome Sci. 2012 Dec 26;10:73. doi: 10.1186/1477-5956-10-73 (PMC3563458; doi:10.1186/1477-5956-10-73)
Supplement: Additional file 1 — MALDI-TOF MS identification of potato leaf proteins regulated by wounding, potato beetle feeding or aphid phloem sap feeding. [file 1477-5956-10-73-S1.pdf]

Additional file 1 MALDI-TOF MS identification of potato leaf proteins regulated by wounding, potato beetle feeding or aphid phloem sap feeding

Spot	Accession number[1]	Identification	p*I* (Exp.[2]/Theor.[3])	M_r (Exp./Theor.)	Sequence coverage (%)	MOWSE score	Matched peptides (m/z)
104	AAM52206	*ATP synthase beta subunit*	5.5/5.1	45.8/53.6	22	79	1490.67<br>2678.16<br>1045.53<br>1201.59<br>1328.57<br>1601.70<br>1617.66<br>2313.89<br>1431.57
304	CAA70392	*RuBisCo large subunit fragment*	9.0/6.5	26.6/44.6	17	52	1450.76<br>1451.76<br>1793.71<br>1794.78<br>1561.78<br>1467.76
426	P58518	*Aspartic protease inhibitor 3*	8.6/8.6	24.6/18.9	32	72	1518.63<br>1348.62<br>1053.53<br>2061.91
444	P12372	*Photosystem I reaction center subunit II. chloroplast precursor*	9.1/9.7	24.4/23.0	30	106	1682.76<br>1175.62<br>1294.66<br>1003.50<br>1527.77<br>906.45<br>888.44
451	Q41229	*Photosystem I reaction center subunit IV B. chloroplast precursor*	9.4/9.7	24.4/15.2	27	62	975.61<br>1003.51<br>875.40<br>2660.25<br>2271.06
469	P58518	*Aspartic protease inhibitor 3*	9.0/8.6	24.3/18.9	32	52	1518.69<br>1348.67<br>1053.57<br>2062.01

[1] NCBInr database.

[2] Experimental p*I* and *M*r values were estimated with the ImageMaster 2D Elite program (GE Healthcare), using Bio-Rad Broad Range molecular standards.

[3] Theoretical p*I* and *M*r values were calculated with the Expasy 'PeptideMass' algorithm (http://ca.expasy.org/tools/pi_tool.html).
