# Supplementary material for: Wounding, insect chewing and phloem sap feeding differentially alter the leaf proteome of potato, Solanum tuberosum L
Source: Proteome Sci. 2012 Dec 26;10:73. doi: 10.1186/1477-5956-10-73 (PMC3563458; doi:10.1186/1477-5956-10-73)
Supplement: Additional file 2 — Ion trap MS/MS identification of potato leaf proteins regulated by wounding, potato beetle feeding or aphid phloem sap feeding. [file 1477-5956-10-73-S2.pdf]

Additional file 2 Ion trap MS/MS identification of potato leaf proteins regulated by wounding, potato beetle feeding or aphid phloem sap feeding

**Spot**	**Accession number[1]**	**Identification**	**p*I* (Exp.[2]/Theor.[3])**	**M_r (Exp./Theor.)**	**MOWSE score**	**Matched unique peptides**
15	*EST537399*	*EST similar to a subtilase (NP_199378)*	8.8/9.4	92.6/85.1	58	SPPGYAVDIVP
76	*EST396117*	*EST similar to a protein disulfide isomerase (Q9XF61)*	4.7/4.8	53.1/57.1	80	EADGIVSYVK<br>AAQILSQNDPPVVLAK
72	P08824	*Rubisco subunit binding-protein alpha subunit*	4.5/4.8	51.752.5	90	TNDSAGDGTTTASVLAR<br>GILNVAAIK
349	Q9THX6	*Putative L-ascorbate peroxidase. chloroplast precursor*	9.0/8.2	25.6/37.9	67	LTLYDAIK<br>STFIASAISK<br>STFIASAISK
396	O24385	*Cysteine protease inhibitor 7*	4.7/8.6	25.0/20.1	62	LCVDETVWKVNDEELVVTGGNVGNENDIFK<br>VNDEELVVTGGNVGNENDIFK
445	P12372	*Photosystem I reaction center subunit II, chloroplast precursor*	9.4/9.7	23.1/22.9	211	EGVGQNFR<br>KEQCLALGTR<br>VFPNGEVQYLHPK<br>EQIFEMPTGGAAIMR
450	CAA45723	*Aspartic protease inhibitor*	9.0/7.5	19.6/23.7	82	YNSDVGPSGTPVR
461	P12354	*Photosystem I reaction center subunit IV. chloroplast precursor*	9.4/10.0	24.4/13.4	73	ESYWYK<br>TRYPVVVR<br>TRYPVVVR<br>YPVVVR
464	S10721	*Cathepsin D inhibitor*	5.3/7.8	24.2/20.8	48	YNSDVGPSGTPVR
504	CAA41439	*Pathogenesis-related protein P2*	8.2/8.5	19.1/16.0	153	LDTNGLGYQR<br>VTNTGTGTQETVR

[1] NCBInr database.

[2] Experimental p*I* and *M*r values were estimated with the ImageMaster 2D Elite program (GE Healthcare), using Bio-Rad Broad Range molecular standards.

[3] Theoretical p*I* and *M*r values were calculated with the Expasy 'PeptideMass' algorithm (http://ca.expasy.org/tools/pi_tool.html).
